# Low molecular weight heparins use in pregnancy: a practice survey from Greece and a review of the literature

**DOI:** 10.1186/s12959-019-0213-9

**Published:** 2019-12-04

**Authors:** E. Papadakis, A. Pouliakis, Α. Aktypi, A. Christoforidou, P. Kotsi, G. Αnagnostou, A. Foifa, E. Grouzi

**Affiliations:** 1 Hemostasis Unit-Hematology Department Papageorgiou Hospital, Thessaloniki Ringroad 56403 Nea Efkarpia, Thessaloniki, Greece; 2 2nd Department of Pathology, National and Kapodistrian University of Athens, “ATTIKON” University Hospital, Rimini 1 Haidari, Athens, Greece; 3 OLYMPION General Clinic, Volou-Patras, 26443 Patras, Greece; 4 University Hospital of Alexandroupolis, Dragana Site 68100 Nea Chili, Alexandroupoli, Greece; 5 Blood Transfusion Unit, National Ref. Centre for Congenital Bleeding Disorders, Hemostasis Unit, Laiko General Hospital, Ag. Thoma, 17 11527 Athens, Greece; 6 Head of Transfusion Service and Clinical Haemostasis, Henry Dunant Hospital Center, Mesogion 107, 115 26 Athens, Greece; 7 IASO, General Maternity and Gynecology Clinic, 37-39, Kifissias Avenue, 151 23 Maroussi, Athens, Greece; 8 ”St Savvas” Oncology Hospital, Alexandras Avenue 171, 11522 Ambelikipoi, Athens, Greece

**Keywords:** Low molecular weight heparin, Pregnancy, Venous thromboembolism, Pregnancy complications

## Abstract

**Background:**

Use of LMWH in pregnancy is not only limited to VTE management, but it extends, to the management of vascular gestational complications and the optimization of IVF pregnancies despite the lack of concrete scientific evidence. In this context, we conducted the present study aiming to gain insights regarding the use of LMWH during pregnancy and puerperium. We recorded indication for use, diagnostic work-up as well as the safety and efficacy of the treatment, trying to elucidate the clinical practice in our country.

**Methods:**

We analyzed data regarding 818 pregnant women received LMWH during 2010–2015.Our cohort had a median age of 33.9 years and a BMI of 23.6.There were 4 groups: those with a history of VTE [Group-A: 76], those with pregnancy complications [Group-B: 445], those undergoing IVF [Group-C: 132] and those carrying prothrombotic tendency (thrombophilia, family history of VTE, other) [Group-D: 165]. Mean duration of LMWH administration was 8.6 ± 1.5 months. Out of the total number, 440 received LMWH in fixed prophylactic dose, 272 in higher prophylactic-weight adjusted dose and 106 in therapeutic dose. Moreover, 152 women received in addition low-dose acetylsalicylic acid (ASA). 93.8% of pregnancies were single and 6.2% were multiple ones. Live births occurred in 98.7% of pregnancies.

**Results:**

Anticoagulation was efficacious and well tolerated. Seventeen VTE events were recorded; 7 of them antepartum and 10 postpartum. No major bleeding events were observed while 13 clinical relevant non-major bleeding events were recorded. Regarding gestational vascular complications, 28 IUGR events were recorded, as well as 48 cases of preterm labor of which 12 were concomitant with IUGR (25%). Six early pregnancy losses were recorded; there were 3 fetal deaths and 3 cases of pre-eclampsia/eclampsia.

**Conclusions:**

LMWHs are used extensively during pregnancy and puerperium in Greece for VTE treatment and prophylaxis and for a variety of other indications as well. Although the drug has been shown to be both safe and efficacious, its use for some indications has no proven scientific evidence. In order to clearly define the role of LMWHs in pregnancy, beyond thromboprophylaxis, large prospective studies are required, which could be based on the conclusions of this study.

## Introduction

It is well known that pregnancy alters the haemostatic system into a hypercoagulable state, which increases throughout pregnancy and is maximal around term. These physiological changes are important for minimizing intra-partum blood loss, but they entail an increased risk of thromboembolism during pregnancy and the post-partum period [1]. For the mother, this risk begins from the time of conception and continues well into the postnatal period, with recent data suggesting that the risk could extend to 12 weeks postpartum [2].

The pro-coagulant state of pregnancy could also contribute to the occurrence of gestational vascular complications (GVCs) (pre-eclampsia, placental abruption, fetal growth restriction (FGR), late and recurrent early miscarriage, intrauterine death and stillbirth), especially in the presence of acquired or inherited thrombophilia [3–5]. There is some evidence suggesting that placental thrombosis could play a role in the pathogenesis of pregnancy loss [6]. On the other hand, other GVCs, such as pre-eclampsia or FGR, have been suggested at least partly due to placental insufficiency, possibly as a result of inappropriate coagulation activation [7].

Anticoagulation with low molecular weight heparins (LMWHs) is a well-established antithrombotic practice for primary and secondary thromboprophylaxis during pregnancy. There has been evidence that heparin and its derivatives could exert a beneficial effect in preventing gestational vascular complications [3, 8]. However, the published data on the role of LMWH were obtained mostly from women with thrombophilia [9]. Low dose aspirin (ASA) and LMWH have proven their effectiveness in increasing live birth rates in the setting of gestational antiphospholipid syndrome. However, their use in the context of inherited thrombophilia and pregnancy complications is less well established.

There is increasing evidence in favor of the use of heparin in women with pregnancy complications mediated by the placenta, selected by previous pregnancy outcome and not by thrombophilic defect [10]. Due to their excellent safety record, LMWHs have been offered to women at high risk of an adverse pregnancy outcome in advance of scientific evidence. The administration of LMWHs in the prevention of pre-eclampsia and small for gestational age (SGA) fetuses is based on biological plausibility and extrapolation from antiphospholipid syndrome [11].

An increasing number of women undergoing assisted reproductive technology (ART) receives LMWHs due to its possible role in increasing the possibility of a successful implantation of the developing embryo in in vitro ART, through modulating a wide variety of proteins involved [12]. It has been suggested that heparins can improve the apposition of the blastocyst and interfere with the apoptosis occurring during the implantation stage of a pregnancy [13]. Two systematic reviews [12, 14] found that the administration of LMWH may increase clinical pregnancy and live birth rates in women undergoing in vitro fertilization (IVF) or intra cell sperm injection (ICSI); authors concluded that, due to the wide heterogeneity of protocols used and the small sample size of women randomized, these results need to be further confirmed in ‘ad hoc’ studies.

In this context, in an attempt to elucidate the clinical practice in our country, we conducted the present cohort study aiming to gain insights regarding the use of LMWHs during pregnancy and puerperium, describing the indications for use, the diagnostic work-up as well as the safety and efficacy of the treatment.

## Patients and methods

A multicenter, retrospective study that addressed the issue of LMWH use in pregnancy in Greece, was performed including pregnant women receiving LMWH for prophylaxis either due to personal history of thromboembolic events, mainly venous thromboembolism (VTE) (group A), or history of GVCs, with the majority of them being less than 3 early pregnancy losses, (group B), or because they were undergoing IVF (group C). A number of women who received LMWH due to family history of VTE, thrombophilia and other, not specified reasons, were also included (Group D). Participants were recruited from seven Hematological Centers all over Greece. The following data were collected for each participant: age, BMI, indication for using LMWH, type and dose of LMWH (fixed-prophylactic dose, higher prophylactic – weight adjusted or therapeutic dose) according to RCOG guidelines, as well as thrombophilia factors [FV Leiden, FII mutation, LAC, antiphospholipid antibodies (APLA) (lupus anticoagulant and/or anticardiolipin and/or β2-glycoprotein-1 antibodies), antithrombin (AT), protein C (PC) and protein S (PS) concentrations]. In addition, low dose ASA (80–100 mg) use was assessed. High risk thrombophilia was defined as the presence of AT deficiency, compound heterozygosity for FV Leiden and FII mutations or homozygosity for FV Leiden or FII mutations and PC and PS deficiencies [15]. All pregnant women receiving thromboprophylaxis were eligible to be included and no exclusion criteria were applied. This was a retrospective study, data were retrieved from women’ medical records and missing data were retrieved via additional contact.

All women were followed-up until the end of puerperium (6 weeks after birth) in order to monitor safety and efficacy of anticoagulation recording any thrombotic or bleeding events. We tracked down VTEs and superficial thromboses objectively confirmed during gestation and puerperium and we noticed any bleeding episode during the same period and classified it according to the definitions as proposed by the ISTH [16, 17]. Data for adverse events that would led to discontinuation or modification of treatment were recorded. Furthermore we recorded any gestational vascular complication and the pregnancy outcome.

## Statistics

Statistical analysis was performed by the SAS for Windows 9.4 software platform [18] (SAS Institute Inc., NC, U.S.A.). Demographic and clinical/prognostic data of the patients at baseline were described with numerical and categorical summary statistics. Statistics were expressed as the mean value along with the standard deviation (SD). In addition, for the sake of completeness, the median value and the values for 25 and 75% quartiles were also reported. Comparisons between two or more groups for the categorical parameters were performed by means of the chi-square test [19]. For the parameters expressed in numerical form (such as the women’s age, their BMI, the duration of treatment with LMWH or ASA, etc.) normality was not always ensured, therefore, non-parametric tests were preferred; more specifically the Kruskal-Wallis test was applied [18]. The significance level (*p*-value) was set to 0.05, thus statistically significant difference between the parameters compared for the groups under study was for *p* < 0.05.

## Results

In total 818 women (mean age 33.9 SD ± 4.9 years) that used LWMH during pregnancy and puerperium were studied. 76 (9.3%) women used LMWH due to personal history of VTE (Deep Vein Thrombosis (DVT), Pulmonary Embolism (PE)) (group A), 445 (54.4%) used LWMH due to pregnancy (early or late) complications (group B), 132 (16.1%) used LMWH after undergoing IVF (group C) and 165 (20.2%) used LMWH for other reasons (group D). The Baseline Characteristics of all patients are presented in Table 1 (and in more details in Table 4 in Appendix), while the summary and comparison of the parameters studied for each group are presented in Table 2 (and in a higher detail in Table 5 in Appendix).

**Table 1:** Baseline Characteristics of the cohort/all patients

Characteristic	*N* = 818
Age (mean ± SD)	33.9 ± 4.9
BMI (mean ± SD)	24.5 ± 4
No. of fetuses at observed gestation (N, %)	
1	767 (93.8)
≥ 2	51 (6.2)
Delivery by CS (N, %)	644 (78.7)
Reason for enrolling in the study	
Group A: History of VTE (DVT/SVT/Arterial thrombosis/Arterial Ischemia)	76 (9.3%)
Group B: History of Pregnancy complications	445 (54.4%)
Group C: IVF	132 (16.1%)
Group D: Other reasons	165 (20.2%)
Mean Duration of LMWH (months) (mean ± SD)	8.6 ± 1.5
Concomitant Use of ASA (N, %)	152 (18.6)
Mean Duration of ASA (months) (mean ± SD)	6.2 ± 2.7

**Table 2:** Baseline characteristics and studied parameters of the four groups along with statistical comparison

	Group A *N* = 76	Group B *N* = 445	Group C *N* = 132	Group D *N* = 165	*p*-value*
Age (mean ± SD)	33.0 ± 4.3	33.5 ± 4.6	37.2 ± 5.1	32.5 ± 4.4	<.0001
BMI (mean, SD)	25.0 ± 4.3	24.4 ± 3.9	24.7 ± 3.8	24.4 ± 4.3	0.2607
No. of foetuses at the observed gestation (N. %)					
1	73, 96.1%	434, 97.5%	102, 77.3%	158, 95.8%	<.0001
≥ 2	3, 4.0%	11, 2.5%	30, 22.7%	7, 4.2%	
Mean Duration of LMWH (months)	8.7 ± 1.7	8.7 ± 1.3	8.7 ± 1.7	8.3 ± 1.6	<.0001
ASA Duration (months) (N of patients)	6.7 ± 2.8 (*N* = 11)	6.1 ± 2.4 (*N* = 79)	5.5 ± 2.8 (*N* = 39)	7.9 ± 2.0 (*N* = 19)	0.0068
Fixed Prophylactic Dose	34.2%	58.9%	50%	52.1%	<.0001
Weight Adjusted prophylactic dose	21.1%	32.4%	38.6%	37.0%	
Therapeutic dose of LMWH	44.7%	8.8%	11.4%	10.9%	
Concomitant Use of ASA	14.5%	18.2%	30.3%	12.1%	0.0006
Caesarian	80.3%	79.7%	91.7%	65.5%	<.0001
Live Birth	97.4%	99.1%	97.0%	99.4%	0.1632
High risk Thrombophilia (positive cases)	25%	10.1%	9.9%	10.3%	0.0018
APA status (total successful tests *N* = 363) (% positive cases within group)	29.6%	29.1%	27.1%	20.4%	0.6264

*p-value is for Kruskal-Wallis test for numerical parameters and for x-square test for categorical parameters

The LMWH compounds that were administered were the following: tinzaparin (innohep®) to 651 women (79.6%, CI: 76.7–82.2%), enoxaparin (Clexane®) to another 140 (17.1%, CI: 14.7–19.8%) and Bemiparin (Ivor®) to 27 (3.3%, CI: 2.3–4.8%) of pregnant women. The mean duration of LMWH administration was 8.6 ± 1.5 months. Among them 440 (53.8%, CI: 50.4–57.2%) received LMWH in fixed prophylactic dose, 272 (33.3%, CI: 30.1–36.6%) received higher prophylactic LMWH dose and 106 (13.0%, CI: 10.8–15.4%) received a therapeutic LMWH dose. Moreover, 152 (18.6%, CI: 16.1–21.4%) women received concomitantly low-dose ASA.

In our cohort, live births were recorded in 807 (98.7%, CI: 97.6–98.7%) pregnancies. Anticoagulation during pregnancy was efficacious and well tolerated. One allergic effect on injection site required intervention. Seventeen VTE events were recorded; 7 (0.8%) of them antepartum and 10 (1.2%) postpartum. Interestingly, no major bleeding events were observed while 13 (1.6%) clinically relevant non major (CRNM) bleeding events were recorded (Table 3). The majority of bleedings [11] were observed antepartum, 10 of them were vaginal blood dripping and 1 was an epistaxis episode. All of them were self-limited but were managed with temporary withhold of anticoagulant treatment, and no-dose adjustment was necessary. Regarding postpartum bleeding we observed 2 episodes. One was CRNM bleeding of the gastric varices in a woman with paroxysmal nocturnal hemoglobinuria and portal vein thrombosis. In her case no transfusion was necessitated but we withhold LMWH and we reduced for 72 h the dose by 50%. The second postpartum bleeding was surgical bleeding from the caesarian section site that was managed by omission of 1 dose of LMWH. One can wonder how we did not observe any major postpartum hemorrhages. That can be attributed to the fact that nearly all women underwent planned cesarean section and LMWH was withhold for 24–48 h. Another possibility could be that the Obstetricians were meticulous since they were coping with high risk pregnancies. All in all we did not record any major postpartum hemorrhage (according to ISTH definition) and no transfusion was needed peripartum. One pregnant developed painful skin rash in her first week of LMWH treatment which was managed with a switch to another LMWH compound, albeit the 30% risk of cross-reactivity. Eventually she underwent an uneventful pregnancy and delivery.

**Table 3:** Events recorded in the cohort and in each of the four groups

	Group A *N* = 76	Group B *N* = 445	Group C *N* = 132	Group D *N* = 165	Total *N* = 818	p*
VTE [VTE postpartum]	3 (3.9%) [1 (1.3%)]	3 (0.7%) [3 (0.7%)]	0 [1 (0.8%)]	1 (0.6%) [5 (3.0%)]	7 (0.8%) [10 (1.2%)]	0.0008
Bleeding	1 (1.3%)	6 (1.3%)	5 (3.8%)	1 (0.6%)	13 (1.6%)	0.151
Gestational vascular complications	12 (15.8%)	37 (8.3%)	23 (17.4%)	16 (9.7%)	88 (10.8%)	0.0064
IUGR	2 (2.6%)	13 (2.9%)	8 (6.1%)	5 (3.0%)	28 (3.4%)	0.8784
Preterm Labor	7 (9.2%)	19 (9.1%)	12 (9.1%)	10 (6.1%)	48 (5.9%)	
Fetal Death	1 (1.3%)	1 (0.2%)	1 (0.8%)		3 (0.4%)	
Early pregnancy loss/abortion	1 (1.3%)	3 (0.7%)	2 (1.5%)		6 (0.7%)	
Pre-eclampsia/eclampsia	1 (1.3%)	1 (0.2%)		1 (0.6%)	3 (0.4%)	

* *p*-value is for chi-square test

Regarding GVCs, 28 (3.4%) intrauterine growth restriction (IUGR) events were recorded, as well as 48 (5.9%) cases of preterm labor – of which 12 were concomitant with IUGR (25%). Six early (< 10 weeks of gestation) pregnancy losses were recorded (0.7%); there were 3 fetal deaths (0.4%) and 3 cases of pre-eclampsia/eclampsia (0.4%).

As was expected, women **in Group A** more often received LMWH at higher doses (67.1%, *p* < .0001) and had a higher percentage of known high risk thrombophilia (25%, *p* = 0.0018), (Table 2). In **Group B,** the largest group of our cohort, the main reason of LMWH administration was early pregnancy losses (*N* = 396 women, 89%). In more detail the major pregnancy complications considered to include these women in group B (see Table 4 in Appendix) were early pregnancy loss (89%), fetal death (6%), IUGR (2%) and eclampsia/pre-eclampsia (2%). Notably, 27% of these women had simultaneously additional history (secondary reasons), such as: retrograde pregnancy (9%), early pregnancy loss and fetal death (3%). Concerning abnormal pregnancy loss, 66% of women had one incident, 22% two incidents and 12% three or more incidents. That was the group in which the majority of women (58.9%) received fixed prophylactic doses of LMWH. In **Group C**, women that received LMWH for IVF optimization, the mean age (37.2 ± 5.1 years) was higher (*p* < .0001) and multiple pregnancy was observed more often (22.7%, *p* < .0001). Furthermore, the vast majority of them (91, 7%) delivered by caesarian section (CS). In **Group D**, a quite heterogeneous cluster, the mean duration of LMWH use was the shortest among all the groups; this was also the group where the higher percentage of vaginal delivery (VD) (34.6%, *p* < .0001) was noted (Table 5 in Appendix).

Within the population under study investigation for the presence of APLA was performed in 363 women (44.4%), out of whom 100 were found positive and 263 negative for APLA. Considering the APLA status in relation to ASA treatment, within our sample, it was found that 53 women out of the 263 with negative APA were treated with ASA (20.2% of the normal APLA population) and 39 women out of the 100 with abnormal APLA status were treated with ASA (39% of the number of women who had tested positive for APLA). Therefore, about twice as many women who tested positive for APLA were simultaneously treated with ASA compared to women who tested negative for APLA (OR: 2.5, 95% CI: 1.5–4.2, *p* = 0.0002). Furthermore the highest percentage of positive APLA was found in the women of Group B who received a therapeutic dose of LMWH (*p* < .0024, Table 6 in Appendix).

In our study cohort, from the baseline characteristics per dosage used it seems that a higher mean age at enrollment, a higher BMI and a presence of high risk thrombophilia were the key drivers for the administration of higher doses (Table 6 in Appendix). Also, there was an association between the personal history of VTE and the dose of LMWH received. Bleeding events, antepartum or postpartum, were not correlated (*p* = 0.82) with higher LMWH doses (Table 7 in Appendix).

Although pregnancy complications were noted in 10.8% of pregnancies only 1.1% of them resulted in fetal loss. It is worth mentioning that the most frequently observed complication was preterm labor in 5.9% of the cases (Table 3).

## Discussion

VTE in pregnancy is an important cause of maternal morbidity and mortality in developed countries. In addition to hemostatic changes occurring during normal pregnancy, several risk factors, including hereditary and acquired thrombophilia, have been identified. Pregnant women are 4 to 5 times more likely to develop VTE than non-pregnant women of a similar age [20]. The components of Virchow’s triad (hypercoagulability, venous stasis and vascular damage) are all affected during pregnancy until the postpartum period [21]. Increases in coagulation factors and decreases in natural anticoagulants during pregnancy lead to a hypercoagulable state. Venous stasis occurs as a result of a diminution in venous return caused by the pressure from the gravid uterus on the iliac veins and vena cava [22]; trauma to the venous system could occur in the course of vaginal delivery (VD) and the risk can be exacerbated by cesarean section (CS). In a recent meta-analysis, the risk of VTE was four times greater following CS than following VD; this seemed independent of other VTE risk factors and was greater following emergency CS than following elective CS [23].

Apart from hereditary thrombophilia certain conditions have been associated with an increased risk of pregnancy related VTE. These include a previous history of thrombosis, antiphospholipid syndrome, lupus and other co-morbidities [24]. Other independent risk factors are age (older than 35 years), null parity, multiple gestation, obesity, smoking and immobility - all these factors represent an 1.5–2-fold increase in the risk [24, 25]. More recently, in pregnancies following IVF, several observational studies [26–29] have reported a higher risk of VTE, independently of the occurrence of ovarian hyper-stimulation syndrome (OHSS) compared to the spontaneous pregnancies. However, in these women, OHSS represents the main factor involved in the VTE occurrence, with a 100-fold increase in risk [26, 30].

Therefore, a careful evaluation, using a validated numerical risk assessment model, of all known preexisting, pregnancy-related and transient risk factors in both antepartum and postpartum periods is crucial to identify moderate−/high-risk women who could benefit from antithrombotic prophylaxis. The RCOG guidelines on antenatal and postnatal thromboprophylaxis [15] recommends a documented risk assessment for VTE in early pregnancy or pre-pregnancy. The assessment needs to be repeated if the woman is hospitalized or in the case of other intercurrent problems occur; and in the intrapartum or peripartum phase as well. These guidelines take into consideration the risk associated with intercurrent problems, obstetric factors and transient risk factors for VTE.

Also, depending on the level of risk it is recommended that, if the decision is made to use antepartum prophylaxis, this should be done from the earliest possible stages of pregnancy, due to the early activation of the hemostatic system [31, 32]. Similarly, as the VTE risk is increased during the first 6–12 weeks postpartum [2], prophylaxis should be extended until 6 weeks after delivery [15, 33, 34]. In most cases the benefits of anticoagulation outweigh its risks.

LMWHs represent the anticoagulant of choice for VTE prophylaxis and treatment in pregnancy, with a clear consensus among the guideline documents reviewed [15, 35, 36]. Compared with UFH, LMWH has a better bioavailability, longer plasma half-life, more predictable dose–response, and improved safety profile with respect to osteoporosis and HIT -heparin induced thrombocytopenia- [15, 35]. As far as the breast-feeding phase is concerned, LMWH as well as UFH and oral anticoagulants (Vitamin K Antagonists (VKA) - not DOACs) have proven safety in breast-feeding women, due to their limited transfer into breast milk [37]. Although LMWHs represent the anticoagulant of choice for VTE prophylaxis in pregnancy [38], the question of optimal dosage and molecules to be used or the weight of each risk factor in predicting the recurrence of VTE are only addressed in a limited way in the literature.

Recurrent miscarriage affects 1–2% of pregnant women, and nearly 50% of these women have idiopathic recurrent miscarriages [39]. Apart from VTE, the pro-coagulant state during pregnancy can be involved in the occurrence of GVCs (i.e., early or late pregnancy loss, intrauterine growth restriction, pre-eclampsia, placental abruption, etc.). It has been hypothesized that in some cases, a thrombotic or an inflammatory process could be partly involved in their origin. Inherited thrombophilia [factor V Leiden (FV G1691A), activated protein C resistance (APCR), prothrombin G20210A gene mutation (FII G20210A), protein C (PC) or S (PS) deficiencies or antithrombin deficiency (AT)] have all been studied in epidemiological studies exploring an association with adverse pregnancy outcomes [6]. The association of APLA with adverse pregnancy outcomes has been recognized and included in the *Sapporo* diagnostic criteria for antiphospholipid syndrome (APS) [40].

Antithrombotic drugs, such as heparins or low doses of ASA, have been suggested to prevent the recurrence of GVCs. Unfortunately, there is a paucity of high-quality evidence from randomized trials in this field, and current recommendations are based on observational studies or evidence gathered from studies in the non-pregnant population.

In a recent review [38] it is concluded, as an expert opinion, that ASA is effective in preventing GVCs in women at risk for pre-eclampsia and in those with APS. Heparins could also confer benefits to women at risk of GVCs (early pregnancy loss in APS, intrauterine fetal death in APS, intrauterine fetal death associated with inherited thrombophilia, pre-eclampsia, small for gestational age newborn, pregnancy loss after an ART attempt) and/or pregnancy-related VTE.

ART has been widely used in couples with fertility problems. According to RCOG, IVF is considered a transient risk factor, thus women with an IVF pregnancy and three other risk factors should be considered for thromboprophylaxis with LMWH starting in the first trimester [15]. In a Norwegian case–control study [41], it was shown that there is an additive effect when ART is performed after multiple pregnancies and a Swedish study showed that IVF increases the risk of VTE by a factor of four and the risk of PE by a factor of seven in the first trimester compared to natural conception [27].

Many studies have investigated the effects of low-dose ASA or LMWH to improve ART outcomes. The biological plausibility of antithrombotic prophylaxis may be represented by a beneficial effect in counteracting existing or developing at risk pro-thrombotic conditions. However, the data are controversial. It has been shown that heparin and its derivatives have a beneficial effect on implantation. Heparins play a role in embryonic implantation and placentation, and contribute to the development of a normal pregnancy. This effect is achieved through the interaction of heparins with coagulation factors, anticoagulation proteins, their effect on the expression of adhesion molecules, matrix degrading enzymes and trophoblast phenotype and apoptosis - all important components in the process of embryonic implantation and placentation. In recurrent implantation failures (RIF) heparins demonstrated a beneficial effect that could be attributed to the effects of this molecule on enhancing endometrial receptivity and trophoblast invasion due to the regulation of heparin-binding factors, adhesion molecules or inhibition of complement activation [8].A meta-analysis of RCTs showed that in women with ≥3 RIF, the addition of LMWH to IVF/ICSI treatment resulted in a 79% improvement in the Live Birth Rate [42]. In addition, a meta-analysis of observational studies showed a significant increase both in the clinical pregnancy rate (RR: 1.83, 95% CI: 1.04–3.23, *p* = 0.04) and in live birth rate (RR: 2.64, 95% CI: 1.84–3.80, *p* < 0.0001) after IVF/ICSI cycles [43]. Additional results suggest that in the case of women with RIF the use of LMWH may have a beneficial effect in improving pregnancy outcomes especially when the outcome “live-birth” was considered [44, 45]. However, in a recent Greek RCT [46] comparing the effects of the administration of LMWH in sub-fertile patients with two or more unsuccessful IVF/ICSI cycles, no evidence was found in support of the standard addition of LMWH in patients with two or more unsuccessful IVF /ICSI cycles.

The present study enrolled a number of pregnant women (*N* = 818) and was conducted in seven hematologic centers all over the country. In this cohort, we aimed at investigating the efficacy and safety of antithrombotic prophylaxis in pregnant women with a history of VTE, in pregnant women with prior recurrent GVCs, or undergoing IVF. Our aim was to assess the occurrence of thrombosis, as well as, explore the utility of LMWH for the prevention of GVCs, for improving pregnancy outcomes and for improving success rates of ART.

The enrollment of pregnant women was heterogeneous since they were selected on the basis of history of VTE, previous pregnancy or IVF outcome. They were also heterogeneous in terms of risk factors, treatment dosage and preparation. Routine thrombophilia screening had been performed to the majority of pregnant women (87%) albeit it had not been complete in all of them. Investigation for the presence of APLA was carried out in 363 out of the 818 women. Thus, some of these women maybe have been classified as low risk for VTE, although they may actually have belonged to the high risk group.

**In Group A** the use of LMWH during pregnancy and puerperium due to personal history of VTE is well supported by studies and recommended by existing guidelines. As was expected, in our study, this was the group that received higher doses of LMWH more often than the other groups. According to our results thromboprophylaxis was efficacious with 4 (5.3%) DVT events and no fatal event. DVTs in our cohort were mainly noticed (Table 3), *p* < .0008), in Group A, a finding which suggests that personal history of VTE stands out among other thrombotic risk factors in pregnancy. What is quite noticeable is the percentage of high risk thrombophilia in Group A which reached 25% which is well above the average in the Greek population [47]**.**

**In Group B** the evidence in favor of the use of LMWH prophylaxis during pregnancy to prevent recurrent GVCs is supported by limited and conflicting evidence [48] and thus, it is not recommended at present by guidelines with the exception of women with APLA. Most studies in pregnancy were non-randomized and retrospective, with a minority of prospective studies generally limited to small sample sizes. Although the use of LMWH in women with a history of GVCs is not supported by hard evidence, the analysis of Group B shows a live birth rate of 99% while 3 events of fetal loss were recorded (0.7%). On the basis of this finding one could argue that the use of LMWH might have a beneficial effect on pregnancy outcome. On the other hand, the majority of the women in this group were enrolled due to early pregnancy losses. In a considerable percentage of women, LMWHs were prescribed in women with single pregnancy loss which weakens the strength of our results.

**In Group C,** although the causes of IVF failure are not very clear, the data from our study seem to support the use of LMWH in women undergoing IVF, since there was a really high live birth rate with practically no VTE events. Although for the women on IVF and the ones with history of GVCs, there is no clear recommendation, the results from our study suggest that LMWH use in these categories of women is safe and effective even if a fixed prophylactic or a higher dosage scheme is selected. Noticeably, in this group thrombotic risk factors clustering was observed such as older age, (> 6 years more), multiple pregnancy and delivery by CS.

**In Group D**, the inherited thrombophilia by itself does not necessitate thromboprophylaxis during pregnancy and puerperium. Indications for thromboprophylaxis of asymptomatic thrombophilia carriers in pregnancy, vary throughout international guidelines, but they are dependent on the type of thrombophilia (high risk vs low risk) and on family history for VTE.

Recent studies have been consistent with a higher risk of pregnancy-related VTE in women who are antithrombin, protein C or S deficient or who are homozygous for factor V Leiden, the prothrombin gene mutation, or are compound heterozygotes for factor V Leiden and the prothrombin gene mutation [49–52]. Family history by itself, in the absence of an identifiable thrombophilic tendency, is associated with an increased risk of VTE. It may be reasonable to consider cases of high-risk thrombophilic families and of asymptomatic pregnant women with a family history of VTE in a first-degree relative aged under 50 years, where the episode has been unprovoked or provoked by pregnancy, combined oral contraceptive exposure or the presence of a minor risk factor [53, 54]. In light of the above, women should be stratified according to both the level of risk associated with their thrombophilia and the presence or absence of a family history or other risk factors [55].

Although not well documented, over prescription of LMWHs in pregnant women with a history of GVCs represents the current clinical practice throughout our country and beyond. This is a practice not adequately supported by scientific data and guidelines that does not taking into account the costs and the side effects of LMWHs but misconceptionally accepts the usefulness of LMWH in women with GVC history as an axiom. LMWHs are used extensively during pregnancy for a plethora of indications, a considerable majority of which has no proven scientific basis. Since the first studies exploring the use of LMWHs to prevent fetal losses produced encouraging results [56], there is a popular misconception among women, many of whom seem to believe that LMWHs use during pregnancy is ‘the shot that prevents miscarriage’. This belief has inadvertently affected the physicians’ practice and has led to inappropriate prescription of antithrombotics in pregnancy. Out of our cohort of 818 women 160 had a clear indication to receive anticoagulants during pregnancy (history of VTE and gestational APS). In another 75 women (carriers of high risk thrombophilia) the use of LMWH is justified by guidelines - albeit not uniformly. Another relevant finding is the increased co-prescription of ASA with LMWH. In our cohort, while 110 women required ASA administration (classical, gestational APS and pre-eclampsia history), actually 152 women were treated with a combination of ASA and LMWH. In a recent Cochrane analysis [57] ASA did not confer a beneficial effect in studies at low risk of bias when combined with LMWH in women with unexplained recurrent miscarriage (with or without inherited thrombophilia). The effect of anticoagulants in women with unexplained recurrent miscarriage and inherited thrombophilia needs to be assessed in further randomized controlled trials. One could argue though that the population that received LMWH without a clear indication was actually in need of thromboprophylaxis due to increased thrombotic risk. This study resulted in the construction of a scoring model for thrombotic risk factors in pregnancy that is based on the latest RCOG guidelines and is currently available as a prognostic calculus throughout Greece for Physicians. It is a digital VTE-risk assessment tool (PAT-pregnancy associated thrombosis-risks) accessible at www.PATrisks.com. Using PAT-risks we evaluated the women of the present cohort for thrombotic risk retrospectively (unpublished data). It is worth mentioning that a considerable percentage of women treated in Groups B, C and D were already at a high VTE-risk according to PAT-risks and the administration of LMWH may have had the additional benefit of preventing thromboembolic events.

Another worrying conclusion is that in the vast majority of women in our cohort, CS was the preferred delivery method. This is due on the one hand to the fact that CS is a well-controlled method of delivery for women under anticoagulation treatment, and on the other that in our country the rate of women who opt for CS is in any case very high [58, 59]. According to published data this is between 48 and 53%, with a higher percentage observed in women having their first child. This fact is stressed by the WHO who point out that “over half the births in the country occur by CS, putting Greece among countries with the highest CS rates in the world” [60]. Given that all the women were in high risk of bleeding due to anticoagulation, this may well account for such a high rate of CS even though it actually carries greater thrombotic risk than the natural vaginal delivery.

In our study the administration of three different LMWHs was recorded. The popularity of tinzaparin could be explained by scientific evidence and data supporting its use in diverse clinical scenarios in pregnancy necessitating VTE prophylaxis. An international, retrospective study of the safety and efficacy profile of tinzaparin use in pregnancy included 1267 pregnancies, making it the largest report of a single LMWH in pregnancy [61]. The above-mentioned study provided reassuring maternal and fetal outcome information in pregnancies exposed to tinzaparin.

## Conclusions

In conclusion, LMWHs are used extensively during pregnancy and puerperium in Greece for VTE treatment and prophylaxis and for a variety of other indications as well. This study is the first national survey regarding LMWHs use in pregnancy and demonstrates the safety and efficacy of the drug in a high risk pregnancy setting. It is of great concern that over prescription of LMWHs in pregnant women with a history of GVCs, despite the lack of solid scientific data, represents the current clinical practice throughout our country and beyond. The study limitations are its non-controlled, retrospective nature, and the heterogeneity of indications for LMWHs administration. The inappropriate use of these drugs should be prevented by establishing and implementing diagnostic and therapeutic guidelines and providing the necessary education for healthcare professionals. In order to clearly define the role of LMWHs in pregnancy, beyond thromboprophylaxis, large prospective studies are required, which could be based on the conclusions of this study.

## Data Availability

The datasets used and/or analysed during the current study are available from the corresponding author on reasonable request.

## Appendix

### Detailed results of the study

**Table 4:** Baseline Characteristics of the cohort/all patients

Characteristic	*N* = 818
Age (mean ± SD, median, q25-q75)	33.86 ± 4.85, 34, 31–37
BMI (mean ± SD, median, q25-q75)	24.5 ± 4, 23.64, 21.87–26.18
No. of foetuses at observed gestation (N, % and 95 CI)	
1	767 (93.77%, CI: 91.90–95.23%)
≥ 2	51 (6.23%, CI: 4.77–8.10%)
Delivery by CS (N, %)	644 (78.7)
Reason for enrolling in the study	
Group A: History of VTE (DVT/SVT/Arterial thrombosis/Arterial Ischemia)	76 (9.29%, CI: 7.49–11.47%)
Group B: History of Pregnancy complications	445 (54.40%, CI: 50.97–57.78%)
*Early pregnancy losses*	*396 (88.99%, CI: 85.74–91.57%)*
*Pregnancy losses < 3–3 or more*	*348–48*
*IUGR*	*10 (2.25%, CI: 1.23–4.09%)*
*Fetal death*	*28 (6.29, CI: 4.39–8.94%)*
*Pre-eclampsia/eclampsia*	*10 (2.25%, CI: 1.23–4.09%)*
*Preterm labor/placenta abruption*	*1 (0.22%, CI: 0.04–1.25%)*
Group C: IVF	132 (16.14%, CI: 13.78–18.82%)
Group D: Other reasons	165 (20.17%, CI: 17.56–23.06%)
*Family History of VTE*	*73 (44.24%, CI: 36.88–51.96%)*
*Asymptomatic Hereditary Thrombophilia*	*45 (27.27%, CI: 21.05–34.52%)*
*Increased resistance in uterine arteries*	*2 (1.21%, CI: 0.03–4.31%)*
*Reasons not specified*	*45 (27.27%, CI: 21.05–34.52%)*
Mean Duration of LMWH (months) (mean ± SD, median, q25-q75)	8.63 ± 1.49, 9, 9–9.5
Concomitant Use of ASA (N, % and 95 CI)	152 (18.58%, CI: 16.06–21.39%)
Mean Duration of ASA (months) (mean ± SD, median, q25-q75)	6.21 ± 2.56, 7, 3–8

**Table 5:** Baseline characteristics and studied parameters of the four groups along with statistical comparison

	Group A *N* = 76	Group B *N* = 445	Group C *N* = 132	Group D *N* = 165	*p*-value*
Age (mean ± SD, median, q25-q75, N)	32.96 ± 4.26, 32.5, 30–36, *N* = 76	33.54 ± 4.59, 34, 31–36, *N* = 445	37.17 ± 5.1, 37, 34–41, *N* = 132	32.48 ± 4.43, 32, 32–35, *N* = 165	<.0001
BMI (mean, SD)	24.98 ± 4.29, 23.88, 21.85–27.48, *N* = 76	24.38 ± 3.91, 23.51, 21.87–25.95, *N* = 445	24.69 ± 3.75, 24.16, 21.84–26.81, *N* = 130	24.44 ± 4.32, 23.51, 23.51–25.86, *N* = 165	0.2607
No. of foetuses at the observed gestation (N. %)					
1	73, 96.05%	434, 97.53%	102, 77.27%	158, 95.76%	<.0001
≥ 2	3, 3.95%	11, 2.47%	30, 22.73%	7, 4.24%	
Mean Duration of LMWH (months)	8.67 ± 1.7, 9, 9–9.5, *N* = 74	8.73 ± 1.32, 9, 9–9.5, *N* = 445	8.69 ± 1.7, 9, 9–9.5, *N* = 131	8.31 ± 1.61, 9, 9–9, *N* = 162	<.0001
ASA Duration	6.73 ± 2.76, 8, 6–9, *N* = 11	6.1 ± 2.38, 7, 3–8, *N* = 79	5.46 ± 2.79, 7, 3–8, *N* = 39	7.89 ± 1.97, 8, 8–9, *N* = 19	0.0068
Fixed Prophylactic Dose	26, 34.21%	262, 58.88%	66, 50%	86, 52.12%	<.0001
Weight Adjusted prophylactic dose	16, 21.05%	144, 32.36%	51, 38.64%	61, 36.97%	
Therapeutic dose of LMWH	34, 44.74%	39, 8.76%	15, 11.36%	18, 10.91%	
Concomitant Use of ASA (N, % women rceived ASA within the group)	11, 14.47%	81, 18.2%	40, 30.3%	20, 12.12%	0.0006
Caesarian	61, 80.26%	354, 79.73%	121, 91.67%	108, 65.45%	<.0001
Live Birth	74, 97.37%	441, 99.1%	128, 96.97%	164, 99.39%	0.1632
High risk Thrombophilia (positive cases)	19, 25%	45, 10.11%	13, 9.85%	17, 10.3%	0.0018
APA status (total successful tests *N* = 363) (positive cases, % within group))	16, 29.63%	57, 29.08%	16, 27.12%	11, 20.37%	0.6264

* *p*-value is for Kruskal-Wallis test for numerical parameters and for x-square test for categorical parameters

**Table 6:** Baseline characteristics per dosage used for each group

Group A*		Fixed prophylactic dose (*n* = 26)	Higher prophylactic (weight/anti-Xa-adjusted) (*n* = 16)	Full treatment dose (*n* = 34)	*p*-value
	Mean age at enrolment	32.23 ± 4.39, 32, 30–35	33.94 ± 3.94, 32.5, 31–37	33.06 ± 4.31, 33, 30–36	0.5021
	BMI	23.02 ± 2.85, 22.86, 20.76–23.88	26.03 ± 3.62, 25.37, 23.66–28.65	25.98 ± 5.01, 24.45, 21.88–29.38	0.0101
	Mean duration of LMWH use	8.82 ± 1.46, 9.5, 9–9.5	9.17 ± 1.25, 9, 9–10	8.34 ± 1.98, 9, 8–9.5	0.1560
	No of foetuses at the observed gestation	1.04 ± 0.2, 1, 1–1	1.06 ± 0.25, 1, 1–1	1.03 ± 0.17, 1, 1–1	0.8560
	High risk thrombophilia (Positives,% in group)	4, 15.38%	3, 18.75%	12, 35.29%	0.1706
	APA status (successful tests *N* = 54) (N positive/N negative, % in treatment type)	7/22, 31.82%	2/11, 18.18%	7/21, 33.33%	0.6440
	History of VTE				
	Unprovoked	6	5	8	0.4238
	Provoked by combined oral contraceptives	20	11	26	
	Concomitant ASA use (Positives,% in group)	3, 11.54%	3, 18.75%	5, 14.71%	0.8111
Group B*		Fixed dose prophylactic (*n* = 262)	Higher prophylactic (*n* = 144)	Therapeutic (*n* = 39)	*p*-value
	Mean age at enrolment	33.43 ± 4.43, 34, 31–36	34.08 ± 4.75, 34, 31–37	32.31 ± 4.92, 33, 29–36	0.2326
	BMI	23.58 ± 3.19, 23.18, 21.67–24.46	25.45 ± 4.6, 24.94, 21.87–28.28	25.77 ± 4.22, 24.57, 23.18–27.18	<.0001
	No of foetuses at the observed gestation	1.02 ± 0.16, 1, 1–1	1.04 ± 0.2, 1, 1–1	1.03 ± 0.16, 1, 1–1	0.2657
	Mean duration of LMWH use	8.51 ± 1.53, 9, 8–9	9.09 ± 0.87, 9, 9–10	8.86 ± 0.83, 9, 9–9.5	0.0001
	High risk thrombophilia (Positives,% in group)	23, 8.78%	18, 12.50%	4, 10.26%	0.4924
	APA status (successful tests *N* = 196) (N positive/N negative, % in treatment type)	30/126, 23.81%	17/55, 30.91%	10/15, 66.67%	0.0024
	History of early pregnancy loss (Positives,% in group)	228, 87.02%	132, 91.67%	36, 92.31%	0.7271
	History of IUGR (Positives,% in group)	5, 1.91%	4, 2.78%	1, 2.56%	
	History of intrauteral fetal death (Positives,% in group)	20, 7.63%	6, 4.17%	2, 5.13%	
	History of pre-eclampsia/eclampsia	8, 3.05%	2, 1.39%	0	
	History of placenta abruption/preterm delivery (Positives,% in group)	1, 0.38%	0	0	
	Concomitant ASA use (Positives,% in group)	34, 12.98%	41, 28.47%	6, 15.38%	0.0005
Group C*		Fixed dose prophylactic (*n* = 66)	Higher prophylactic (*n* = 51)	Therapeutic (*n* = 15)	*p*-value
	Mean age at enrolment	36.18 ± 4.95, 36, 34–39	38.55 ± 5.01, 38, 35–42	36.87 ± 5.3, 37, 32–42	0.0559
	BMI	23.71 ± 3.71, 22.86, 21.05–24.978	25.48 ± 3.87, 24.83, 23.03–28.604765	26.34 ± 2.27, 26.3, 24.91–27.89	0.0003
	No of foetuses at the observed gestation	1.15 ± 0.36, 1, 1–1	1.27 ± 0.49, 1, 1–2	1.47 ± 0.52, 1, 1–2	0.0278
	Mean duration of LMWH use	8.18 ± 2.12, 9, 8.5–9	9.23 ± 1, 9, 9–10	9.17 ± 0.45, 9, 9–9.5	0.0007
	High risk thrombophilia (Positives,% in group)	5, 7.58%	5, 9.80%	3, 20.00%	0.3456
	APA status (successful tests *N* = 59) (N positive/N negative, % in treatment type)	10/33, 30.30%	6/20, 30.00%	0/6, 0%	0.2885
	Concomitant ASA use (Positives,% in group)	18, 27.27%	20, 39.22%	2, 13.33%	0.1194
Group D*		Fixed dose prophylactic (*n* = 86)	Higher prophylactic (*n* = 61)	Therapeutic (*n* = 18)	*p*-value
	Mean age at enrolment	31.33 ± 4.38, 31, 29–34	33.49 ± 4.35, 34, 31–36	34.61 ± 3.42, 34.5, 32–37	0.0007
	BMI	23.68 ± 2.68, 23.57, 21.87–25.65	24.09 ± 4.26, 23.12, 21.21–25.34	29.3 ± 7.21, 27.59, 24.51–33.13	0.0004
	No of foetuses at the observed gestation	1.02 ± 0.15, 1, 1–1	1.07 ± 0.25, 1, 1–1	1.06 ± 0.24, 1, 1–1	0.4385
	Mean duration of LMWH use	8.15 ± 1.63, 9, 8–9	8.6 ± 1.37, 9, 9–9	8.06 ± 2.17, 9, 9–9	0.0521
	High risk thrombophilia (Positives,% in group)	7, 8.14%	7, 11.48%	3, 16.67%	0.5181
	APA status (successful tests *N* = 54) (N positive/N negative, % in treatment type)	3/26, 11.54%	8/26, 30.77%	0/2, 0%	0.1742
	Family History of VTE (Positives,% in group)	48, 55.81%	21, 34.43%	4, 22.22%	0.0003
	Asymptomatic Thrombophilia	22, 25.58%	21, 34.43%	2, 11.11%	
	Increased resistance in uterine arteries (Positives,% in group)	2, 2.33%	0	0	
	Reasons not specified (Positives,% in group)	14, 16.28%	19, 31.15%	12, 66.67%	
	Concomitant ASA use (Positives,% in group)	7, 8.14%	13, 21.31%	0	0.0136

* *p*-value is a) for Kruskal-Wallis for the numerical parameters or b) chi-square for categorical parameters, numerical parameters are reported as: Mean ± SD, Median, 25% quantile −75% quantile75, categorical parameters are reported as N (positive cases), % of positive cases within the treatment type group

**Table 7:** Events in the total cohort and per group for each dose

Group A		Fixed dose prophylactic (*n* = 26)	Higher prophylactic (*n* = 16)	Therapeutic (*n* = 34)	*p*
	VTE (pre or postpartum)	3, 11.54%	0	3, 8.82%	0.3894
	Bleeding	0	0	1, 2.94%	0.5348
	Adverse pregnancy complications	2, 7.69%	2, 12.50%	8, 23.53%	0.2295
	*IUGR*	*1, 3.85%*	*0*	*1, 2.94%*	*0.5193*
	*Preterm Labor*	*0*	*2, 12.50%*	*5, 14.71*	
	*Fetal Death*	*0*	*0*	*1, 2.94%*	
	*Early pregnancy loss/abortion*	*1, 3.85%*	*0*	*0*	
	*Pre-eclampsia/eclampsia*	*0*	*0*	*1, 2.94%*	
Group B		Fixed dose prophylactic (*n* = 262)	Higher prophylactic (*n* = 144)	Therapeutic (*n* = 39)	*p*
	VTE (pre or postpartum)	1, 0.38%	1, 0.69%	3, 7.69%	0.0002
	Bleeding	5, 1.91%	1, 0.69%	o	0.4462
	Adverse pregnancy complications	18, 6.87%	11, 2.47%	3, 7.69%	0.952
	*IUGR*	*2, 0.76%*	*6, 4.17%*	*0*	*0.315*
	*Preterm Labor*	*12, 4.58%*	*5, 3.47%*	*2, 5.13%*	
	*Fetal Death*	*1, 0.38%*	*0*	*0*	
	*Early pregnancy loss/abortion*	*2, 0.76%*	*0*	*1, 2.56%*	
	*Pre-eclampsia/eclampsia*	*1, 0.38%*	*0*	*0*	
Group C		Fixed dose prophylactic (*n* = 66)	Higher prophylactic (*n* = 51)	Therapeutic (*n* = 15)	*p*
	VTE (pre or postpartum)	0	1, 1.96%	0	0.4492
	Bleeding	2, 3.03%	3, 5.88%	0	0.5199
	Adverse pregnancy complications	8, 12.12%	10, 19.61%	2, 13.33%	0.5226
	*IUGR*	*1, 1.52%*	*4, 7.84%*	*0*	*0.4374*
	*Preterm Labor*	*5, 7.58%*	*5, 9.80%*	*2, 13.33%*	
	*Fetal Death*	*0*	*1, 1.96%*	*0*	
	*Early pregnancy loss/abortion*	*2, 3.03%*	*0*	*0*	
	*Pre-eclampsia/eclampsia*	*0*	*0*	*0*	
Group D		Fixed dose prophylactic (*n* = 86)	Higher prophylactic (*n* = 61)	Therapeutic (*n* = 18)	*p*
	VTE (pre or postpartum)	1, 1.16%	5, 8.20%	0	0.055
	Bleeding	0	1, 1.64%	0	0.4242
	Adverse pregnancy complications	5, 5.81%	6, 9.84%	1, 5.56%	*0.7414*
	*IUGR*	*0*	*1, 1.64%*	*0*	
	*Preterm Labor*	*5, 5.81%*	*4, 6.56%*	*1, 5.56%*	
	*Fetal Death*	*0*	*0*	*0*	
	*Early pregnancy loss/abortion*	*0*	*0*	*0*	
	*Pre-eclampsia/eclampsia*	*0*	*1, 1.64%*	*0*	
All Groups		Fixed dose prophylactic (*n* = 440)	Higher prophylactic (*n* = 272)	Therapeutic (*n* = 106)	*p*
	VTE (pre or postpartum)	5, 1.14%	7, 2.57%	6, 5.66%	0.0151
	Bleeding	7, 1.59%	5, 1.84%	1, 0.94%	0.8226
	Adverse pregnancy complications	33, 7.50%	29, 10.66%	14, 13.21%	0.1218
	*IUGR*	*4, 0.91%*	*11, 4.04%*	*1, 0.94%*	*0.0592*
	*Preterm Labor*	*22, 5.00%*	*16, 5.88%*	*10, 9.43%*	
	*Fetal Death*	*1, 0.23%*	*1, 0.37%*	*1, 0.94%*	
	*Early pregnancy loss/abortion*	*5, 1.14%*	*0*	*1, 0.94%*	
	*Pre-eclampsia/eclampsia*	*1, 0.23%*	*1, 0.37%*	*1, 0.94%*	

## Abbreviations

APCR: Activated protein C resistance
APLA: Antiphospholipid antibodies
APS: Antiphospholipid syndrome
ART: Assisted reproduction techniques
ASA: Acetylsalicylic acid
AT: Antithrombin
BMI: Body mass index
CRNM: Clinically relevant non major
CS: Caesarian section
DVT: Deep venous thrombosis
FGR: Fetal growth restriction
GVCs: Gestational vascular complications
HIT: Heparin induced thrombocytopenia
ICSI: Intracytoplasmic sperm injection
IUGR: Intrauterine growth retardation
IVF: In vitro fertilization
LAC: Lupus anticoagulant
LMWH: Low molecular weight heparin
OHSS: Ovarian hyper-stimulation syndrome
PAT: Pregnancy associated thrombosis
PE: Pulmonary embolism
RCOG: Royal college of Obstetricians and Gynecologists
RIF: Recurrent implantation failures
SD: Standard deviation
SGA: Small for gestational age
UFH: Unfractionated heparin
VD: Vaginal delivery
VTE: Venous thromboembolism
